# Epidemiology of Oral Cancer in Cyprus: A Population-Based Cancer Registry Study and Policy Implications

**DOI:** 10.7759/cureus.94016

**Published:** 2025-10-07

**Authors:** Chrystala Charalambous, Aristomenis Syngelakis, Maria Myrto Solomou, George Pantelas, Smaragda Diamanti

**Affiliations:** 1 School of Dentistry, European University Cyprus, Nicosia, CYP

**Keywords:** cyprus, epidemiology, health policy, oral cancer, risk factors

## Abstract

Objective: Oral cancer is a major global health issue, with prognosis limited by late diagnosis and aggressive behavior. Although Cyprus has reported a low incidence historically, recent increases highlight the need for epidemiological assessment.

Methods: A retrospective population-based study analyzed oral cancer cases recorded from 1998 to 2021. Cases were identified using ICD-10 codes. Demographic variables (sex, age), tumor site, and staging were examined. Crude and age-standardized incidence rates were calculated to assess trends.

Results: A total of 1,070 oral cancer cases were identified (1.4% of all cancers), with a male predominance (688 men (64.3%) and 382 women (35.7%)). Most patients were aged ≥55 years (69.8%), while 29% were <55. Incidence increased over time, especially for cancers of the tongue and oral cavity. Localized disease was the single largest category (38% overall; 46% among cases with known stage), while 6% had distant metastases. Although incidence in Cyprus is below global averages, recent trends reflect those in developed countries.

Conclusions: The rising incidence of oral cancer in Cyprus highlights the need for targeted public health strategies. Initiatives should focus on reducing risk factors, raising awareness among professionals and the public, and improving screening to enhance early detection.

## Introduction

Oral cancer, a subset of head and neck cancers, encompasses malignancies that develop in the tissues of the oral cavity and oropharynx. This includes cancers of the lips, tongue, buccal mucosa, floor of the mouth, hard and soft palate, sinuses, and oropharynx [1,2]. The most common type is oral squamous cell carcinoma (OSCC), which accounts for approximately 90% of cases and originates in the squamous cells lining the oral cavity and throat [1].

Oral cancer is frequently incidentally discovered, and the most common presenting symptoms include dysphagia, odynophagia, otalgia, hoarseness, mucosal irregularities and ulceration, oral or oropharyngeal pain, weight loss, or the presence of an unexplained neck mass [1]. If left untreated, it can invade nearby tissues and metastasize to other parts of the body, most commonly the lymph nodes, significantly impacting survival outcomes [2].

Globally, oral cancer represents a significant public health burden. It is among the 10 most common cancers worldwide [1-3], with over 450,000 new cases diagnosed annually [1,4,5]. Despite advancements in treatment, survival rates remain low due to delayed diagnoses and aggressive disease progression [5]. In Europe, trends show geographic disparities in incidence and mortality rates, with Central and Eastern Europe bearing the highest burden [6,7]. Risk factors such as tobacco use, alcohol consumption, human papillomavirus (HPV) infection, and socioeconomic inequalities contribute to these patterns [7,8].

In Cyprus, oral cancer is comparatively less common but still poses a growing concern [4]. While Cyprus reports relatively low overall cancer mortality rates compared to other European Union (EU) countries, increasing trends in cancer incidence highlight the need for enhanced prevention and early detection efforts [9,10]. The limited awareness among healthcare professionals and the general population about oral cancer risk factors further exacerbates challenges in addressing this disease [11]. The primary aim of this study was to evaluate the epidemiology of oral cancer in Cyprus using population-based cancer registry data. Further aims were to estimate age-standardized incidence rates (ASR) and annual percentage changes (APC) for oral cancer (C00-C14), to describe incidence patterns by sex, subsite, and stage at diagnosis and to discuss the public health and policy implications of these findings. By examining epidemiological trends, risk factors, survival rates, and prevention strategies, this research seeks to inform evidence-based public health policies and improve outcomes for individuals affected by oral cancer.

## Materials and methods

This study is a population-based retrospective observational study of oral cancer in Cyprus for the years 1998 to 2021. The data include all registered cases of oral cancer from the Cyprus Cancer Registry [12], maintained by the Health Monitoring Unit of the Ministry of Health of the Republic of Cyprus. Population denominators were obtained from the Cyprus Statistical Service [13] (mid-year resident population estimates). The analysis included all malignant neoplasms of the lip, oral cavity, and pharynx (International Classification of Diseases, 10th Revision (ICD-10) C00-C14 [14]) diagnosed between 1998 and 2021. We excluded cases among non-residents of the Republic of Cyprus Government Controlled Area, in situ cases, and benign tumors. Death-certificate-only (DCO) cases were included but analyzed separately in sensitivity checks. All cases of oral cancer registered in the database from January 1, 1998, to December 31, 2021, were analyzed. The records were extracted through automated systems from the Cyprus Cancer Registry [12], ensuring accuracy and consistency. Patient confidentiality was strictly maintained throughout the study. Oral cancer cases were identified using ICD-10 codes (Table 1). Key variables such as sex, age, and type of cancer were collected and analyzed to describe the epidemiological characteristics of the disease in the Cypriot population.

**Table 1:** ICD-10 topography codes (C00-C14) for lip, oral cavity, and pharyngeal cancers [14].

C00.0 External upper lip	C06.0 Cheek mucosa
C00.1 External lower lip	C06.1 Vestibule of mouth
C00.2 External lip, NOS	C06.2 Retromolar area
C00.4 Mucosa of lower lip	C06.8 Overl. lesion of unspec. parts of mouth
C00.9 Lip, NOS	C06.9 Mouth, NOS
C01.9 Base of tongue, NOS	C07.9 Parotid gland
C02.0 Dorsal surface of tongue, NOS	C08.0 Submandibular gland
C02.1 Border of tongue	C08.8 Overl. lesion of major salivary gland
C02.2 Ventral surface of tongue, NOS	C08.9 Major salivary gland, NOS
C02.3 Anterior 2/3 of tongue, NOS	C09.8 Overl. lesion of tonsil
C02.9 Tongue, NOS	C09.9 Tonsil, NOS
C03.0 Upper gum	C10.3 Posterior wall of oropharynx
C03.1 Lower gum	C10.4 Branchial cleft
C03.9 Gum, NOS	C10.9 Oropharynx, NOS
C04.8 Overl. lesion of floor of mouth	C11.1 Posterior wall of nasopharynx
C04.9 Floor of mouth, NOS	C11.9 Nasopharynx, NOS
C05.0 Hard palate	C12.9 Pyriform sinus
C05.1 Soft palate, NOS	C13.2 Posterior wall of hypopharynx
C05.2 Uvula	C13.9 Hypopharynx, NOS
C05.8 Overl. lesion of palate	C14.0 Pharynx, NOS
C05.9 Palate, NOS	

Incidence rates were age-standardized by direct standardization to the World Standard Population (Segi-Doll, CI5) (Table 2) [15]. We used five-year age bands and mid-year population denominators from the Cyprus Statistical Service [13]. Age-specific incidence rates were multiplied by the corresponding World Standard weights and summed to yield the age-standardized rate per 100,000 (ASR(W)). Ninety-five percent confidence intervals (95% CI), which represent the range within which the true rate is expected to lie 95% of the time, were calculated using the modified gamma method (Tiwari).

**Table 2:** Age group weights of the World Standard Population (Segi-Doll, CI5) [15]. Weights are expressed as absolute values summing to 100,000 across 18 age groups (0-4 through 85+). These weights were applied in direct standardization to compute age-standardized incidence rates (ASR(W)) per 100,000 population. The World Standard Population (Segi-Doll, CI5) is recommended for comparability in international cancer incidence reporting.

Age group (years)	Weight
0-4	12,000
5-9	10,000
10-14	9,000
15-19	9,000
20-24	8,000
25-29	8,000
30-34	6,000
35-39	6,000
40-44	6,000
45-49	6,000
50-54	5,000
55-59	4,000
60-64	4,000
65-69	3,000
70-74	2,000
75-79	1,000
80-84	500
85+	500
Total	100,000

Crude incidence rates and age-standardized rates (ASR(W)), calculated as described above using the World Standard Population, were summarized by sex, age group, calendar year, and cancer site. Temporal trends in crude rates and ASR(W) were assessed using Poisson regression models with the calendar year as a continuous predictor and the log of the population as an offset, providing APC estimates with 95% CIs. Differences in stage distribution between age groups and sexes were evaluated using the chi-squared test for independence (or Fisher’s exact test where expected counts were <5). Comparisons of site-specific incidence by sex were also assessed using the chi-squared test. Statistical analyses were performed using IBM SPSS Statistics (version 26.0; IBM Corp., Armonk, NY, US). All hypothesis tests were two-sided with α = 0.05. ASR(W) were computed by direct standardization to the World Standard Population (Segi-Doll, CI5) using five-year bands; 95% CIs were estimated via the modified gamma (Tiwari) method.

Temporal trends in incidence were assessed by modeling annual case counts with Poisson regression, using the calendar year as a continuous predictor and the log of the mid-year population as an offset. This approach provided estimates of the APC with corresponding 95% CIs. Model fit was evaluated, and where overdispersion was detected, a negative binomial model was applied. Analyses were stratified by sex and by major anatomical site groups (tongue, oral cavity, and oropharynx) to explore site-specific trends.

Thirteen cases (1.2%) had unknown age and were retained in the overall totals but excluded from age-specific and age-standardized incidence calculations. Stage information was missing in 185 cases (17%); these were reported as “unknown” in stage tables/figures and excluded from percentage denominators when describing stage distribution. We refrained from interpreting temporal changes in early detection where the proportion of unknown stage varied substantially by year.

Categorical variables are reported as n (%), and continuous variables as mean ± SD or median (IQR) as appropriate. Trends in annual incidence were assessed by Poisson regression with log(population) offset; where over-dispersion was detected, we used negative binomial regression. Comparisons of proportions (e.g., stage by sex/age; site by sex) used chi-squared tests. We report test statistics (Wald χ² or z for trend; χ² for independence; exact tests where used) with p-values and 95% CIs. All tests are two-sided; statistical significance was set at α = 0.05 (we denote p < 0.001 as highly significant).

## Results

**Figure 1:**
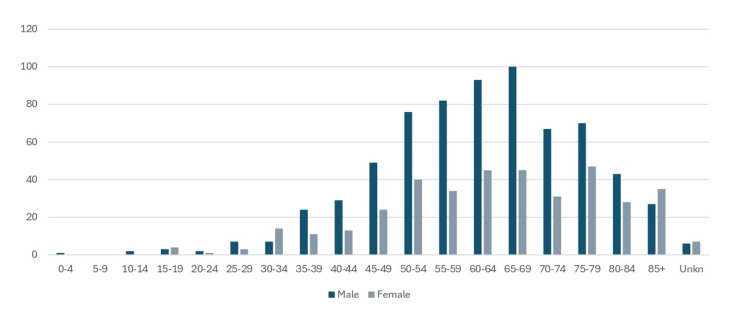
Age distribution of lip, oral cavity, and pharyngeal cancers (ICD-10 C00-C14) by sex, Cyprus, 1998-2021. Values are represented as case counts (n), excluding in situ cases and non-residents. This shows the number of cancers diagnosed in each age group for men and women. Source: Cyprus Cancer Registry [12]

The dataset includes 1,070 cases of oral cancer (C00-C14) registered in the Cyprus Cancer Registry from 1998 to 2021. Of these cases, 688 (64.3%) were men and 382 (35.7%) were women. The age distribution of cases shows the highest number of cases in the 65-69 age group (13.6%), followed by the 60-64 age group (12.9%) and the 75-79 age group (10.9%). The lowest number of cases occurred in the youngest age groups, with one case in the 0-4 age group and two cases in the 10-14 age group; 1.2% cases had an unknown age (Figure 1). All totals and percentages are internally consistent; unknown values (e.g., age, sex, and stage) are reported separately where applicable.

**Figure 2:**
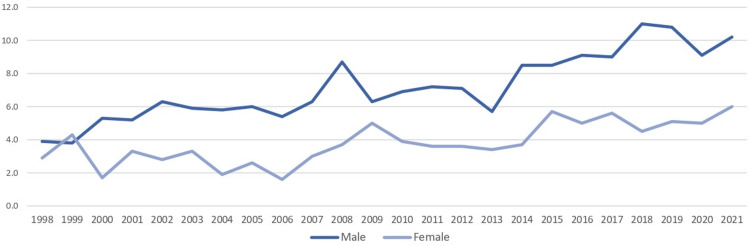
Crude incidence rates of C00-C14 cancers per 100,000 population, Cyprus, 1998-2021. Denominators based on mid-year resident population estimates from the Cyprus Statistical Service. Rates exclude in situ cases and cases among non-residents (±95% CI). Statistical significance was set at p < 0.001. Error bars indicate 95% confidence intervals (95% CI: the range in which the true rate is expected to fall 95% of the time). “Crude rate” means the raw rate in the population, without adjusting for age structure. Source: Cyprus Statistical Service [13]

Crude incidence rates per 100,000 population showed a general upward trend for both sexes over the years. For men, crude rates increased from 3.9 in 1998 to 10.2 in 2021, while for women, rates rose from 2.9 in 1998 to 6.0 in 2021 (Figure 2).

**Figure 3:**
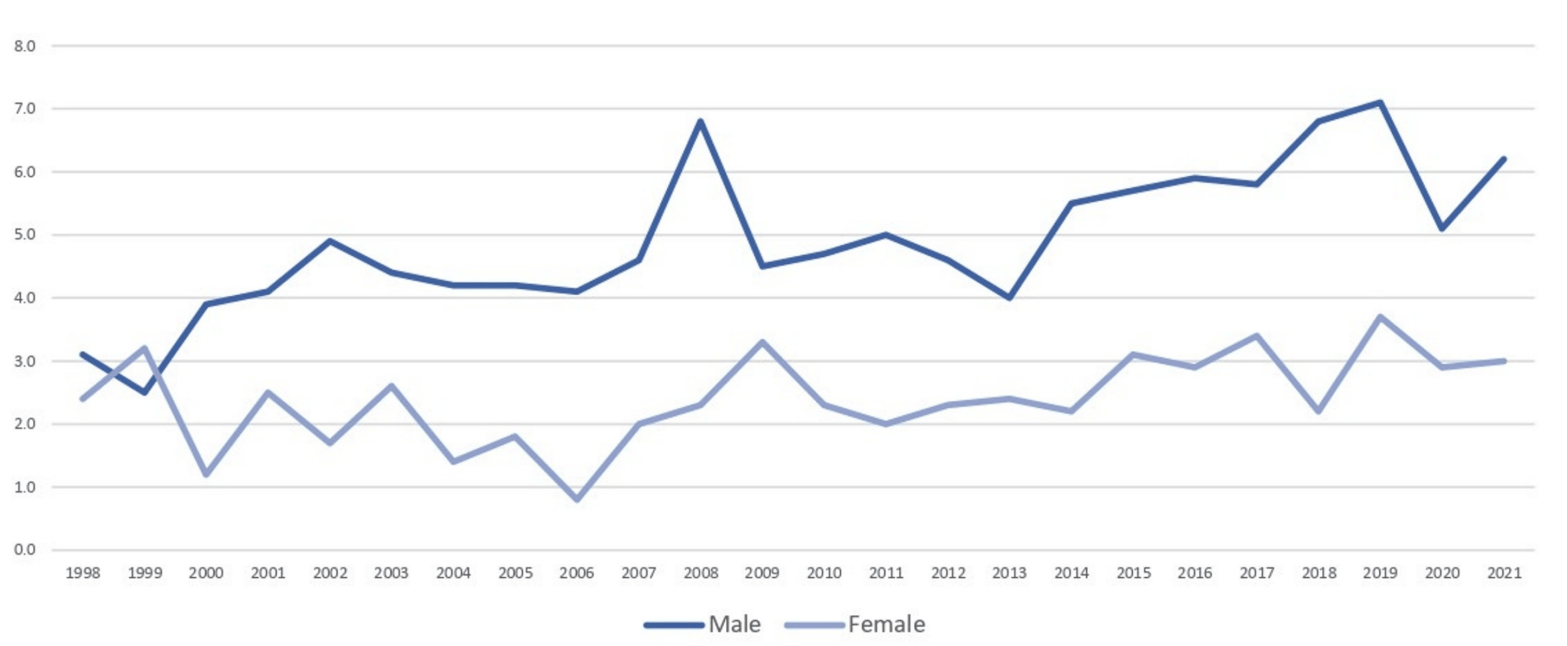
Age-standardized incidence rates (ASR(W) per 100,000, standardized to the World Standard Population (Segi-Doll, CI5), 5-year age bands) for C00-C14 cancers, Cyprus, 1998-2021. Error bars represent 95% confidence intervals (Tiwari modified gamma method). Rates exclude in situ cases and cases among non-residents (±95% ASR). Statistical significance was set at p < 0.001. “Age-standardized” means the rate has been adjusted so differences are not simply due to an older or younger population. Source: World Standard Population (Segi-Doll, CI5) [14]

ASR(W) also reflected an increasing trend for men, rising from 3.1 in 1998 to 6.2 in 2021, whereas for women, ASR(W) fluctuated but ended at a similar level as earlier years, at around 3.0 (Figure 3).

**Figure 4:**
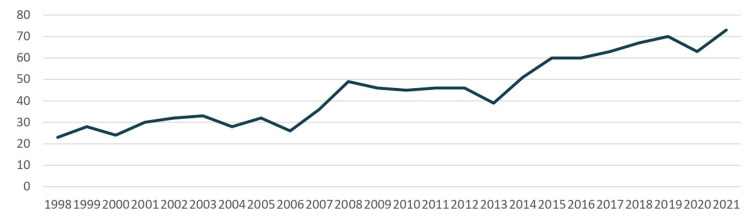
Annual number of C00-C14 cancer cases, Cyprus, 1998-2021. Values are case counts (n), excluding in situ cases and non-residents. This figure shows the total number of new cancers diagnosed each year.

**Figure 5:**
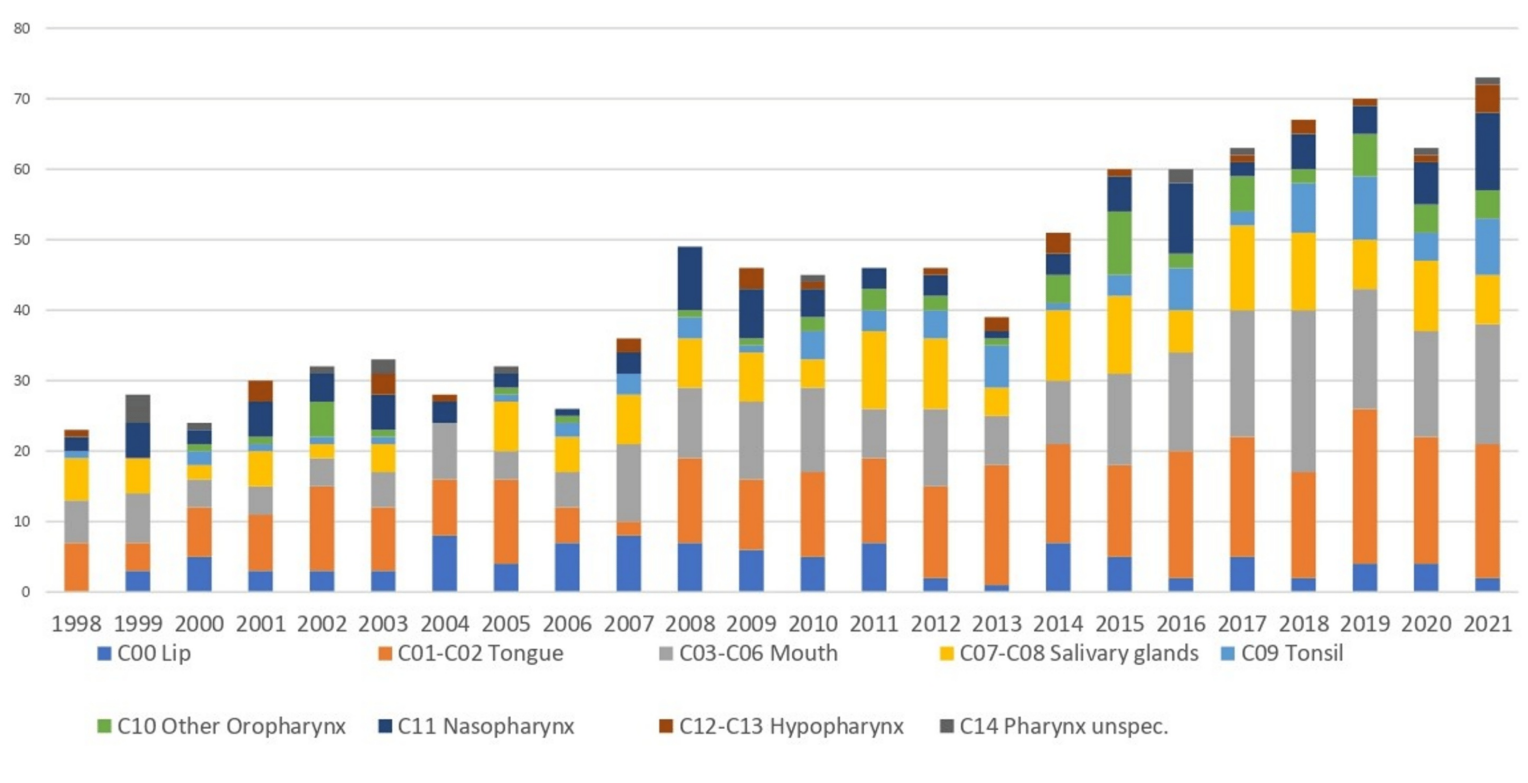
Crude incidence rates of C00-C14 cancers per 100,000 population, Cyprus, 1998-2021. Denominators are mid-year resident population estimates from the Cyprus Statistical Service. Rates exclude in situ cases and non-residents. Error bars show 95% CI. Statistical significance was set at p < 0.001. Source: Cyprus Statistical Service [13] CI: confidence interval

The highest annual number of cases was recorded in 2021, with 73 incident cancers (C00-C14). In that year, a restricted grouping (C00-C10) accounted for 57 of 4,309 new cancer diagnoses nationwide (1.3%). The total number of cases increased over time, with notable increments observed after 2007. From 1998 to 2021, the incidence of oral cavity and pharyngeal cancers (C00-C14) increased significantly, with an APC of +5.0% (95% CI 4.0-5.9, p < 0.001 (Figures 4, 5).

**Figure 6:**
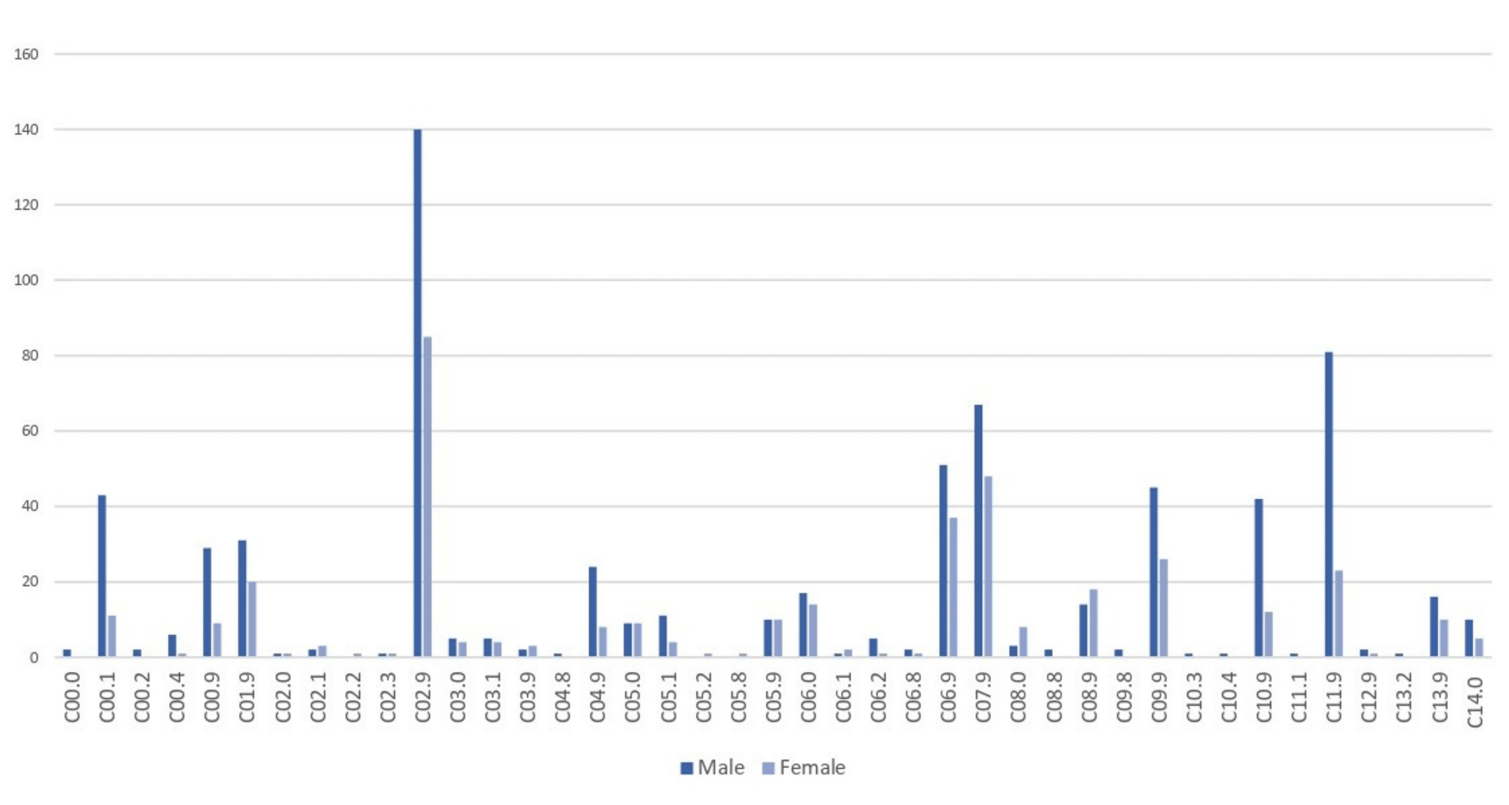
Distribution of C00-C14 cancer cases by anatomical subsite (topography), Cyprus, 1998-2021. Values are case counts (n), excluding in situ cases and non-residents. Subsites include lip, tongue, other oral cavity, and oropharynx. This illustrates which parts of the mouth and throat were most often affected.

The anatomical distribution of oral cancer cases revealed that the tongue (C01-C02) was the most commonly affected site, with a total of 286 cases (26.7%) (175 men and 111 women). This was followed by cancers of the mouth (C03-C06), with a total of 242 cases (22.6%) (143 men and 99 women), and salivary glands (C07-C08), with a total of 160 cases (14.9%) (86 men and 74 women). Other notable sites included the oropharynx (C10) with 56 cases (44 men and 12 women), nasopharynx (C11) with a total of 105 cases (82 men and 23 women), and the tonsils (C09), with a total of 73 cases (47 men and 26 women) (Figure 6).

**Figure 7:**
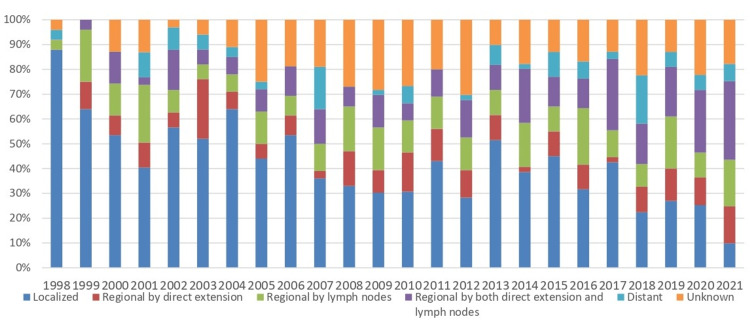
Stage at diagnosis of C00-C14 cancers, Cyprus, 1998-2021. Stage was defined using SEER Summary Stage categories [16] (localized, regional, distant, and unknown). Percentages (%) exclude cases with an unknown stage unless otherwise specified. Statistical significance was set at p < 0.001. “Localized” means confined to the site of origin, “regional” means spread to nearby tissues or lymph nodes, and “distant” means metastasis to distant organs.

Regarding the stage at diagnosis, SEER Summary Stage categories [16] showed localized disease accounted for the largest proportion of cases, with a total of 404 cases (37.8%). Regional disease involving lymph nodes or direct extension accounted for a combined total of approximately 417 cases (39%), distributed as follows: regional by direct extension alone (Stage II) had 106 cases (9.9%), regional by lymph nodes alone (Stage III) had 150 cases (14%), and regional by both direct extension and lymph nodes (Stage IV) had 161 cases (15%). Distant metastases were recorded in only 64 cases (6%), while the stage was unknown for the remaining 185 cases (17%). The temporal trends in stage distribution showed that localized disease constituted a higher percentage of diagnosed cases over the years, while advanced stages at diagnosis remained at low percentages (Figure 7).

## Discussion

This study represents the first comprehensive investigation into the epidemiology of lip, oral cavity, and pharyngeal cancers (C00-C14) in Cyprus.

Incidence

In 2020, Cyprus recorded an age-standardized cancer incidence rate of 639 cases per 100,000 population, exceeding the EU average of 569 [10]. Among men, prostate cancer accounts for 31% of new cases, followed by lung cancer (13%) and colorectal cancer (11%) [10]. For women, breast cancer was the most prevalent (34%), with thyroid cancer (10%) and colorectal cancer (7%) also being significant [10]. In 2021, cancers of the lip, oral cavity, and oropharynx (C00-C10) accounted for 57 of 4,309 incident cancers in Cyprus (1.3%).

Globally, the incidence of oral cavity and pharyngeal cancers has generally declined since the mid-1990s, largely due to reductions in tobacco consumption [17,18]. Not all regions perceive this trend. In some developed countries, cases have increased, often linked to lifestyle changes and a rising prevalence of HPV-related cancers [17]. Europe reflects these diverse patterns, with incidence trends varying significantly by region. Western European nations have reported declines, primarily due to reduced smoking rates, while countries like the United Kingdom have observed increases in specific subtypes, such as oropharyngeal cancer, which is predominantly associated with HPV infections [18,19].

According to the literature, oral cancer is most commonly diagnosed in the fifth-sixth decade of life, with the majority of cases occurring in older individuals [3]. Our research findings are consistent with this trend, as 747 cases (69.8%) in our study were diagnosed in patients aged ≥55 years old. However, while the majority of cases occurred in this age group, it is important to highlight that a significant proportion of patients (310 cases (29%)) were diagnosed before the age of 55. This underscores the need for awareness and early detection efforts across a broader age range. Moreover, this also confirms the growing trend observed in recent years, where oral cancer increasingly affects younger individuals [3].

In contrast to the global decline, oral cancer incidence in Cyprus has shown a rising trend over time, particularly among men [4]. This increase suggests growing exposure to risk factors such as tobacco use, alcohol consumption, and other lifestyle or environmental influences [4,20]. The consistently higher incidence in men compared to women highlights a gender disparity, aligning with global patterns where men are more frequently affected due to higher tobacco and alcohol use [20,21]. These findings have significant public health implications, emphasizing the need for targeted prevention strategies.

When compared to global and European data, Cyprus exhibits a lower but still increasing incidence of oral cancer. In 2020, the global ASR(W) for oral cancer was 8.1 per 100,000 population [17], whereas Cyprus reported approximately 6.2 per 100,000 for men and 3.0 per 100,000 for women by 2021 [4,20]. In 2022, Europe recorded 62,103 new cases, with men comprising 66.9% and an ASR(W) of 6.4, compared to 2.2 for women [7]. While Cyprus rates remain below both global and European averages, the country follows the same upward trend seen worldwide [4,20]. The global increase, however, is more pronounced due to a larger population base and higher exposure to region-specific risk factors, such as betel quid chewing in South Asia [20].

Despite its smaller population, Cyprus’s oral cancer incidence remains significant relative to its size, with 1,070 cases recorded between 1998 and 2021 [17]. The country shares common risk factors with global trends, primarily tobacco and alcohol use, though public health interventions may influence future rates [17,21]. For instance, the introduction of HPV vaccination in 2016 is expected to help reduce HPV-related oral and oropharyngeal cancers in the future, although this effect will only become evident over the coming decades [22]. Continued surveillance and prevention efforts are crucial to addressing this growing public health concern.

An important methodological issue is that the definition of “oral cancer” is not consistent across the literature. Some studies restrict it to C00-C06 (oral cavity only), others extend to C00-C10 (including lip, salivary glands, and oropharynx), while others use the broader C00-C14 grouping that also incorporates nasopharyngeal and hypopharyngeal sites. In our study, the primary analyses were based on C00-C14, consistent with the reporting conventions of the Cyprus Cancer Registry [12] and European Network of Cancer Registries (ENCR) guidelines. For transparency and comparability, we additionally present a subset table restricted to C00-C10, which allows readers to align our findings with studies that apply this narrower grouping. Making these distinctions explicit is important, as risk factors, staging, and trends can differ between oral cavity cancers, HPV-related oropharyngeal cancers, and other pharyngeal sites.

Formal trend analysis confirmed a significant upward trajectory, with a +5% increase per year over the study period. This pace of rise is faster than that reported in several European registries, highlighting a concerning pattern in Cyprus.

Risk factors

In 2021, 35.8% of adults in Cyprus used tobacco, with a difference of 47.8% of men compared to 23.8% of women [23], higher than the global average of 34.9% [23]. The European Union (EU) average for people consuming 20 or more cigarettes per day was 5.9% [24]. In Cyprus, 9.7% of adults smoke more than 20 cigarettes per day [24], which is nearly double the EU average.

The cultural acceptance of smoking in Cyprus, particularly among men, further amplifies this effect [25]. Smoking remains socially acceptable in Cyprus, contributing to its prevalence. Social norms and peer behaviors play a significant role in maintaining high smoking rates, particularly among younger populations [25,26]. The perception that smoking is common can lead individuals to underestimate its risks and continue the habit. Cyprus has implemented several tobacco control policies, including a comprehensive smoking ban in enclosed public places since January 2010 [26].

**Figure 8:**
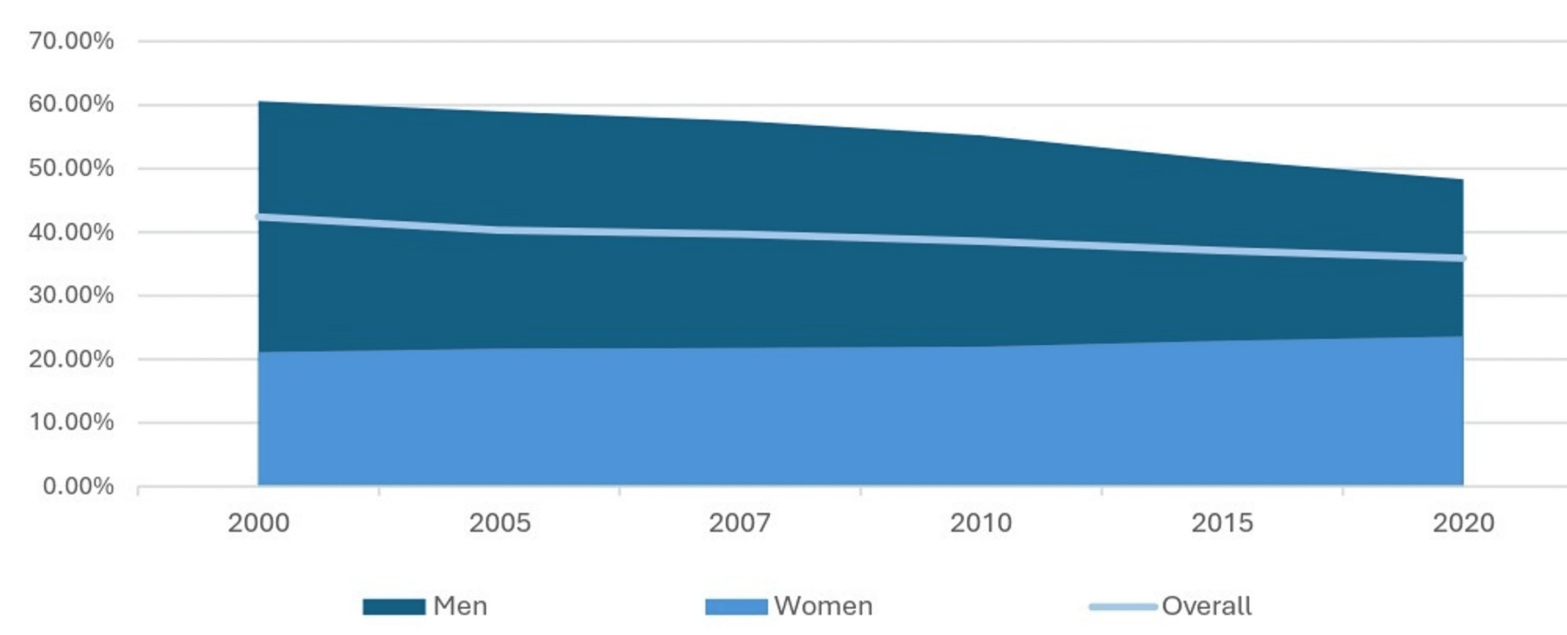
Prevalence of tobacco smoking among persons aged ≥15 years in Cyprus, 1998-2021 (% of population). Source: Cyprus Statistical Service [13]/WHO Global Health Observatory [28]

Analyzing the historical smoking prevalence in Cyprus reveals a notable decline over the past two decades, particularly among men (Figure 8). According to the Global Health Observatory Data Repository [27], in 2000, approximately 60.6% of adult men and 21.1% women were smokers, and by 2015, this figure had changed to 51.4% of male and 22.9% of female smokers, while in 2020, 48.3% of men and 23.6% reported smoking.

The paradox of decreasing smoking rates alongside increasing oral cancer incidence suggests that factors beyond tobacco use may be contributing to the rise in oral cancer cases. These factors could include increased alcohol consumption, HPV infections, dietary habits, and environmental exposures.

One emerging area of concern is the growing popularity of electronic cigarettes, particularly among adolescents of secondary school age. According to findings from the European School Survey Project on Alcohol and Other Drugs (ESPAD) [29], Cypriot students aged 15-16 years rank among the highest in Europe in the use of e-cigarettes and hookah, with daily use reported by 4.6%, well above the European average of 3.1% [29]. However, existing surveillance data provide limited detail by schooling level or socioeconomic background. Understanding the specific factors that contribute to initiation, such as peer influence, accessibility, and school environment, would enable more precisely targeted public health policies and school-based prevention strategies.

Furthermore, based on data from a recent general population survey conducted by the Cyprus National Addictions Authority (NAAC), over half of cigarette smokers (57%) have tried e-cigarettes, a significant jump from 21% in 2019. Although many users have been found to turn to e-cigarettes in an attempt to quit traditional smoking, especially in the 25-44 age group, their long-term effects, particularly in relation to oral cancer, remain insufficiently studied. This rising trend in e-cigarette use could represent an overlooked risk factor and warrants further investigation in the context of the increasing incidence of oral cancer in Cyprus. Additionally, improved diagnostic techniques and greater awareness may lead to more frequent detection of oral cancers, contributing to the observed increase in reported cases.

Alcohol consumption, while less prevalent in Cyprus compared to other EU nations [25], remains a significant risk factor, particularly when combined with smoking, as their synergistic effects increase oral cancer risk [1-3]. In Cyprus, the adult per capita consumption of alcohol for the period of 2003-2005 was 9.3 L [30], less than the 12.2 L that is the average of the WHO European region for that same period. As of 2019, the average adult per capita alcohol consumption in Cyprus was 9.6 L of pure alcohol. This figure was still lower than the EU average of 11.3 L per adult for the same year [27].

The observed increase in incidence, particularly among men and on the lateral tongue and floor of the mouth, due to higher rates of tobacco and alcohol use, is consistent with global trends [1-3,31], while women may have a slightly higher prevalence of cancers in less common sites like the gingiva or palate when risk factors such as HPV are considered [1,31].

A notable difference in the trend seems to be the frequency of lip cancer in Cyprus. Though rare, it represents a distinct subtype influenced by cumulative sun exposure. This highlights the importance of targeted education and sun protection measures for high-risk groups.

While HPV infections are widely linked to oropharyngeal cancers worldwide, data specifically on HPV-related oral cancers in Cyprus remain scarce [10,23,30]. Studies have shown that the global prevalence of HPV in OSCC is approximately 10% in the oral cavity and 42% in the oropharynx, with HPV16 being the most frequently detected genotype, especially in oropharyngeal cases [7,32]. Higher rates of HPV-positive OSCC have been observed in regions such as North America, Northern Europe, and Oceania [7,32]. However, further research is necessary to validate these findings and investigate potential regional differences.

Other lifestyle factors, such as poor diet, physical inactivity, and obesity, also contribute to oral cancer susceptibility, as these factors can indirectly promote carcinogenesis [10]. These findings underscore the critical need for comprehensive public health strategies to address lifestyle and environmental factors that contribute to the disease burden.

A significant clinical concern is the stage at which oral cancers are diagnosed. In Cyprus, the majority of oral cancer cases continue to be diagnosed at early stages, and only a small proportion are diagnosed at advanced stages, indicating that most patients are being identified before the disease progresses. While there has been some variation in stage distribution over recent years, early-stage diagnoses have remained consistently high, reaching 77% in 2021. Importantly, the rate of late-stage diagnoses remains low (6%), and continued efforts to improve early detection are essential.

While tobacco, alcohol, HPV, and emerging exposures such as e-cigarettes are plausible contributing factors, our registry-based design did not capture individual-level exposures. Accordingly, these associations should be interpreted at the ecological level, and targeted etiologic studies are warranted to clarify the role of specific risk factors in Cyprus.

An important constraint of this study is the proportion of cases with missing information (13 unknown age (1.2%), 17% unknown stage). While we presented these separately and excluded them from denominators in stage-specific percentages, residual uncertainty remains. The apparent increase in localized-stage diagnoses should, therefore, be interpreted cautiously, as it may partly reflect variation in staging completeness rather than true improvement in early detection. The absence of this information may be due to incomplete clinical documentation, inconsistencies in diagnostic practices, or challenges in data reporting. Recognizing this issue, efforts are currently being made to regulate the reporting process by law, requiring all healthcare providers to submit staging information to the Health Monitoring Unit. This initiative aims to ensure that staging data are consistently recorded and reported, allowing for more comprehensive and accurate epidemiological analyses in future studies.

The presentation of a new healthcare system in Cyprus in 2019 marked a transformative shift in the country's healthcare, creating a unified environment that integrates both public and private sectors into a single market. This system, which combines elements of a National Health Service and a social health insurance scheme, provides universal coverage and free access to healthcare services for all beneficiaries [33]. Notably, inpatient care, including oral cancer cases, began to be covered under this system in June 2020. A key feature of the new health plan is that it allows patients to choose their doctors and hospitals for treatment. While this flexibility empowers patients and enhances accessibility, it remains to be seen how the system will influence various health indicators, including early detection rates, treatment outcomes, and overall cancer care quality.

Furthermore, it was suggested that, despite the oral cavity's accessibility, there may be a lack of awareness and proactive screening practices among Cypriots, which could hinder early diagnosis [11]. While early detection is crucial for improving survival rates, the same study also indicates that a notable percentage of Cypriots lack adequate information about oral cancer and its prevention [11]. These findings highlight the urgent need for preventive measures and improved early detection strategies to mitigate the growing proportion of late-stage diagnoses and improve long-term survival rates.

Compared to other types of cancer, such as cervical and breast cancer, oral cancer tends to have a higher percentage of diagnoses at earlier stages. This may be attributed to the relatively easy access and visualization of the oral cavity, allowing for earlier detection of suspicious lesions during routine dental check-ups or self-examinations. In contrast, cervical cancer often requires specialized screening methods, such as Pap smears and HPV testing, which may not be regularly utilized by all individuals [34-36]. Similarly, breast cancer, despite advancements in mammography and self-exams, can sometimes remain undetected until it reaches a more advanced stage, particularly in cases of dense breast tissue or aggressive tumor subtypes [35,36]. The visibility of oral cancer lesions facilitates earlier clinical identification, potentially contributing to a higher proportion of early-stage diagnoses compared to cancers that develop in less accessible areas of the body.

The increase in late-stage oral cancer diagnoses in recent years can likely be attributed to several COVID-19 pandemic-related factors such as lockdowns and restricted hospital visits [37]. One major contributor was the healthcare system strain, as resources were largely redirected toward managing COVID-19, leaving non-urgent conditions overlooked. This resulted in delayed referrals and, consequently, more advanced-stage diagnoses. Additionally, increased risk factor exposure during lockdowns, such as higher tobacco and alcohol consumption due to stress [37], could have accelerated disease progression in high-risk individuals. Limited access to dental care also played a critical role, as dentists were unavailable due to clinic closures or reduced services, leading to missed early detections [37]. Lastly, diagnostic challenges and patient delays further compounded the issue, as many individuals experiencing symptoms avoided seeking medical attention due to fear of visiting healthcare facilities, ultimately resulting in delayed diagnoses [37].

Mortality rate

Cancer is the second leading cause of death in Cyprus, with the first being cardiovascular diseases [30]. Even with a higher cancer incidence rate than the EU average, Cyprus recorded the lowest cancer mortality rate in the EU, with 195 deaths per 100,000 population [10,30]. Mortality trends for oral cavity cancers vary significantly across Europe. In 2022, 24,253 deaths from oral cavity cancers were reported, with countries such as Hungary and Russia experiencing some of the highest global mortality rates [7]. Across Europe, mortality trends have evolved differently by gender. While many countries have seen a decline in oral cancer mortality among men, mortality rates among women have slightly increased in recent decades [20]. This rise is primarily attributed to a growing prevalence of smoking among women, highlighting the complex interplay of risk factors influencing these patterns [19,20].

Survival rates and prognosis

The prognosis for oral cancer is influenced by several key factors. Early detection significantly improves outcomes, while late-stage diagnoses often lead to poorer prognoses and increased mortality [7]. The size of the primary tumor plays a significant role, with larger tumors (T2-T4) being associated with worse overall survival [38,39]. Additionally, the extent of recurrent disease is critical, as patients with locoregional recurrence have significantly lower survival rates compared to those with local recurrence [38,39]. Surgical margins also impact prognosis, with close or positive margins after primary tumor resection linked to poorer outcomes [38]. Another factor affecting prognosis is the presence of extranodal extension in affected lymph nodes at the initial diagnosis [39]. Furthermore, the type of primary treatment received, particularly prior radiation therapy and chemotherapy, can influence survival outcomes in recurrent cases [38]. The five-year survival rate in Cyprus stands at 66%, comparable to Finland and Sweden (60%-70%) and slightly below the United States (68%) [40]. Survival is notably poorer among older patients, often due to comorbidities that complicate treatment and recovery.

Prevention and early detection strategies

A study conducted in 2012 found that only 53% of Cypriot participants were aware of oral cancer. While approximately half of the respondents reported having some knowledge of the condition, only 32.5% were aware of available screening options for oral cancer [11]. However, more recent data from the Cyprus Cancer Registry in 2019 [12] showed a notable improvement, with overall awareness of oral cancer rising to 74% and awareness of screening possibilities increasing to 58.7% of the population. These figures are comparable to findings from similar studies conducted in other European countries [41]. The significant increase in public awareness reflects the effectiveness of the measures and initiatives implemented over recent years, and it is anticipated that this positive trend will continue, with even higher awareness levels expected in the coming years.

Preventive measures are essential for reducing the burden of oral cancer. Public health initiatives emphasize lifestyle modifications, such as smoking cessation, reducing alcohol consumption, adopting a diet rich in fruits and vegetables, and increasing physical activity [10,30].

Cyprus has implemented several legislative measures to reduce smoking, recognizing its significant role as a risk factor for oral cancer and other chronic diseases. The Health Protection (Tobacco Control) Law of 2002 to 2009, supplemented by the Health Protection (Tobacco Control) Laws of 2004 and 2017, introduced stricter regulations on tobacco sales, advertising, and smoking in public places. While these policies have contributed to an overall decline in smoking rates, the reduction has not been as substantial as expected (Figure 8). This indicates that while legislative efforts have made some impact, enforcement challenges, cultural attitudes, and strong social acceptance of smoking may have limited their effectiveness.

HPV vaccination programs are also promoted as a critical measure to prevent virus-associated oropharyngeal cancers [42,43]. Cyprus incorporated the HPV vaccine into its National Immunization Program in 2016. The latest available data on vaccination coverage from 2016 to 2020 indicate that the percentage of fully vaccinated 15-year-old girls was 54% in 2018, rising to 59% in 2019 and 64% in 2020 [10].

Awareness campaigns play a central role in educating the general population about risk factors and early symptoms, as well as encouraging regular dental check-ups for the identification of precancerous or early-stage lesions [44-46]. Community-based campaigns and individual oral healthcare providers can play a pivotal role in early detection through opportunistic screening and patient education, particularly as public knowledge and misinformation strongly influence care-seeking behavior [36]. According to studies [44,45], public awareness campaigns can lead to increased recognition of oral cancer symptoms and promote earlier consultations with healthcare providers [45].

In Cyprus, various awareness and prevention activities are implemented to address oral cancer at both public and individual levels. The first week of December is designated as Oral Cancer Awareness Week, which emphasizes the importance of self-examination and regular dental visits as key components for early detection. During this week, mobile dental units offer free examinations to the public, facilitating accessible check-ups aimed at encouraging early detection. The Ministry of Health also organizes educational campaigns that focus on risk factors such as smoking, alcohol consumption, and HPV, as well as early symptoms of oral cancer. These campaigns often include the distribution of informational materials like brochures and pamphlets to educate the public. Local health authorities and non-governmental organizations (NGOs) collaborate to conduct community events that further enhance awareness. These events may include workshops and health fairs that provide interactive sessions aimed at educating attendees about oral cancer prevention, as well as presentations addressing risk factors and the importance of recognizing early symptoms. Additionally, continuing education programs are implemented to enhance the knowledge and skills of healthcare providers, particularly dentists, regarding oral cancer detection. This training includes how to conduct effective screenings and guidance on educating patients about self-examination techniques and preventive measures.

This study has several strengths, including the use of population-based registry data spanning 24 years and the application of internationally comparable age standardization methods. Nonetheless, limitations should be acknowledged. First, a proportion of cases were DCO, which may impact the completeness of incidence estimates and stage distribution. Staging completeness also varied by year, and the proportion of unknown stage must be considered when interpreting early-diagnosis trends. Furthermore, our discussion of tobacco use among adolescents relied on overall prevalence estimates; disaggregated data by age group and schooling level were not available, limiting granularity. These limitations suggest that while the overall incidence trends are robust, some year-to-year variation may reflect changes in registry practice or healthcare system dynamics rather than solely underlying disease risk.

Health surveillance and future steps

Cyprus benefits from a population-based cancer registry system, which collects and analyzes comprehensive data on cancer trends. This system enables the evaluation of incidence rates and survival outcomes over time. However, the declining proportion of reported early-stage cases suggests potential underuse of screening tools. Additionally, external audits by the European Commission's Health Monitoring Unit highlight the need for continuous improvement in health data systems and coding accuracy. Future efforts should prioritize integrating prevention, early diagnosis, and treatment within a cohesive framework. Such a framework should emphasize individualized care for older patients, who often experience unfavorable survival outcomes due to late-stage diagnoses and comorbidities. Addressing these gaps through a combination of policy-driven interventions and community-based efforts will be essential.

## Conclusions

Oral cancer in Cyprus presents a distinct case study within the global and European context, reflecting both its relatively rare occurrence and significant health challenges. Strengthening prevention efforts, alongside fostering early detection through education and advanced screening methods, offers promising avenues to reduce the disease burden. Investment in public health campaigns, advanced diagnostic tools, and healthcare professional training will be critical in addressing current challenges and potential future increases in cancer incidence. Given that oral health is influenced by multiple factors, including diet, hygiene, smoking, alcohol use, stress, and trauma, a multisectoral collaboration is needed. Since these risk factors are common to several other chronic diseases, adopting a common risk factor approach is more rational than a disease-specific strategy.

Legislative measures play a crucial role in oral cancer control by targeting key risk factors. Policies like smoking bans, taxation on tobacco and alcohol products, restrictions on advertising, and mandatory health warnings have proven effective in reducing exposure to carcinogens and encouraging behavioral change. However, beyond legislation, a broader upstream approach is essential for long-term oral cancer prevention. This includes public health education, improving access to healthcare services, integrating oral cancer screenings into routine check-ups, and addressing social determinants of health.
